# An enhanced antioxidant strategy of astaxanthin encapsulated in ROS-responsive nanoparticles for combating cisplatin-induced ototoxicity

**DOI:** 10.1186/s12951-022-01485-8

**Published:** 2022-06-10

**Authors:** Jiayi Gu, Xueling Wang, Yuming Chen, Ke Xu, Dehong Yu, Hao Wu

**Affiliations:** 1 Department of Otolaryngology-Head and Neck Surgery, Shanghai Ninth People’s Hospital, Shanghai Jiao Tong University School of Medicine, Shanghai, China; 2 Ear Institute, Shanghai Jiao Tong University School of Medicine, Shanghai, China; 3 Shanghai Key Laboratory of Translational Medicine on Ear and Nose Diseases (14DZ2260300), Shanghai, China; 4 Materdicine Lab, School of Life Sciences, Shanghai University, Shanghai, China

**Keywords:** ROS-responsive, Poly(propylene sulfide), Astaxanthin, Cisplatin-induced ototoxicity

## Abstract

**Background:**

Excessive accumulation of reactive oxygen species (ROS) has been documented as the crucial cellular mechanism of cisplatin-induced ototoxicity. However, numerous antioxidants have failed in clinical studies partly due to inefficient drug delivery to the cochlea. A drug delivery system is an attractive strategy to overcome this drawback.

**Methods and results:**

In the present study, we proposed the combination of antioxidant astaxanthin (ATX) and ROS-responsive/consuming nanoparticles (PPS-NP) to combat cisplatin-induced ototoxicity. ATX-PPS-NP were constructed by the self-assembly of an amphiphilic hyperbranched polyphosphoester containing thioketal units, which scavenged ROS and disintegrate to release the encapsulated ATX. The ROS-sensitivity was confirmed by ^1^H nuclear magnetic resonance spectroscopy, transmission electron microscopy and an H_2_O_2_ ON/OFF stimulated model. Enhanced release profiles stimulated by H_2_O_2_ were verified in artificial perilymph, the HEI-OC1 cell line and guinea pigs. In addition, ATX-PPS-NP efficiently inhibited cisplatin-induced HEI-OC1 cell cytotoxicity and apoptosis compared with ATX or PPS-NP alone, suggesting an enhanced effect of the combination of the natural active compound ATX and ROS-consuming PPS-NP. Moreover, ATX-PPS-NP attenuated outer hair cell losses in cultured organ of Corti. In guinea pigs, NiRe-PPS-NP verified a quick penetration across the round window membrane and ATX-PPS-NP showed protective effect on spiral ganglion neurons, which further attenuated cisplatin-induced moderate hearing loss. Further studies revealed that the protective mechanisms involved decreasing excessive ROS generation, reducing inflammatory chemokine (interleukin-6) release, increasing antioxidant glutathione expression and inhibiting the mitochondrial apoptotic pathway.

**Conclusions:**

Thus, this ROS-responsive nanoparticle encapsulating ATX has favorable potential in the prevention of cisplatin-induced hearing loss.

**Graphical Abstract:**

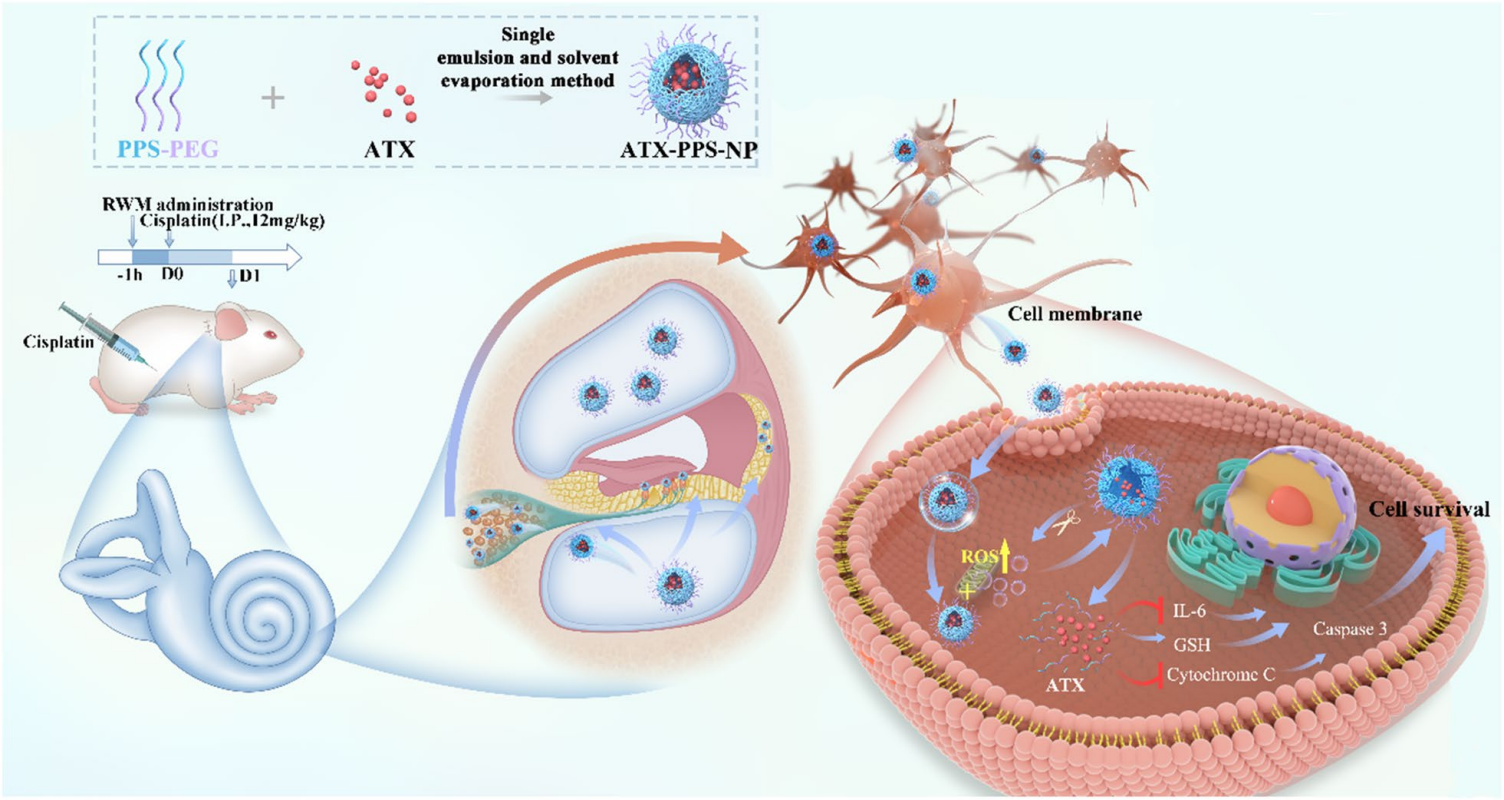

**Supplementary Information:**

The online version contains supplementary material available at 10.1186/s12951-022-01485-8.

## Introduction

To date, the precise mechanism underlying cisplatin-induced ototoxicity is still unclear. However, accumulation of reactive oxygen species (ROS) has been documented as a critical mediator in cisplatin-induced ototoxicity, involved in many pathological processes. In the cochlea, cisplatin upregulates the expression of NADPH oxidase 3 and xanthine oxidase, two major sources of ROS [1–3]. ROS then facilitate the imbalance between ROS and the antioxidant system by depleting antioxidant molecules, including superoxide dismutase, glutathione peroxidase, and catalase [4–6], which is capable of inducing cochlear lipid peroxidation by increasing concentrations of toxic chemicals, such as malondialdehyde or 4-hydroxynonenal (4-HNE) [7–9]. Besides, excessive ROS opens calcium-permeable channels in sarcoplasmic/endoplasmic reticulum membranes and plasma membranes [10], leading to an increase in cytosolic calcium levels and eventual apoptotic and autophagic cell death [11].

A wide range of therapeutic molecules including antioxidants (apocynin, vitamin C), anti-inflammatory (curcumin, epigallocatechin-3-gallate), anti-apoptotic (pifithrin‐α) compounds, have been applied in preclinical studies due to their preventative and restorative effects. Of these compounds, antioxidants exhibit application prospects. Astaxanthin (ATX), a xanthophyll carotenoid, has strong antioxidant capacity by scavenging free radicals, quenching singlet oxygen, enhancing antioxidant enzymes and inhibiting lipid peroxidation [12]. Meanwhile, it is associated with maintaining the mitochondrial redox state and functional integrity against oxidative stress [13]. However, due to severe restriction of the blood-labyrinth barrier, lack of sufficient drug concentrations in the inner ear often exist after intravenous injection or oral intake (systemic administration) [14]. Even after intratympanic injection (local administration), ATX can hardly penetrate the round window membrane (RWM) and enter the inner ear due to its hydrophobicity and instability. In our previous study, ATX first demonstrated its otoprotective effect against cisplatin-induced ototoxicity with the aid of lipid-polymer hybrid nanoparticles (LPN) [15]. Therefore, inner ear drug delivery strategies have attracted much attention due to their contribution in allowing drug access to the inner ear, improving cochlear drug concentrations and ultimately strengthening drug efficacy [16, 17].

Recently, increasing attention has been paid to the development of stimuli-responsive nanoparticles, which realize the accurate spatiotemporal control of drug release and minimize toxicity by avoiding damage to non-target sites [18]. The stimuli that activate the responsiveness of nanomaterials can either be endogenous (redox, pH, enzyme) or exogenous (light, heat, magnetic field and ultrasound) [19]. Notably, ROS-responsive drug delivery systems in endogenous pathological conditions have been extensively studied [20–23]. According to the literatures, ROS-responsive systems include polysulfides, polyselenides, polythioketals, polyoxalates, oligoproline- and catechol-based materials [24]. Poly(propylene sulfide) (PPS), as a type of polysulfide, can be successively oxidized to sulfoxide by hydrogen peroxide (H_2_O_2_) and to sulfone by hypochlorite [25]. The two-step reaction results in a large change in polarity and increases water solubility, consequently leading to an extensive disintegration of PPS and control of drug release. As reported, Poly(propylene sulfide)-poly(ethylene glycol) (PPS-PEG) has been used for drug delivery in a broad spectrum of diseases, including cancers, diabetes and inflammation-related injuries [26–28]. Recently, ROS-responsive nanoparticles encapsulating berberine have been developed and applied in noise-induced hearing loss [29].

In this study, we fabricated a novel ROS-responsive/consuming nanoparticle system loaded with the antioxidant ATX (ATX-PPS-NP) and confirmed its ‘on demand’ spatiotemporal release in vitro and in vivo. This system combined the ROS-consuming effect of PPS-PEG and the antioxidant efficacy of ATX. The protection against cisplatin-induced ototoxicity was verified in HEI-OC1 cells, cochlear explants and guinea pigs, where antioxidant/anti-inflammatory/anti-apoptotic processes were involved. ATX-PPS-NP eventually reduced the loss of spiral ganglion neuron (SGNs) and prevented hearing loss in animal models of cisplatin-induced hearing loss. Thus, providing a novel strategy for the prevention of cisplatin-induced hearing loss.

## Materials and methods

### Materials

PPS-PEG (molecular weight [MW] 54 kDa) and FITC-conjugated PPS-PEG (FITC-PPS-PEG, MW 54 kDa) were purchased from Xi’an Ruixi Biological Technology Co., Ltd (Xi’an, China), and successful synthesis was confirmed by ^1^H nuclear magnetic resonance (^1^H-NMR) spectra. As shown in Additional file 1: Fig. S1, FTIR spectra illustrated an absorption peak at 1024 cm^−1^ after treating PPS-PEG with 30% H_2_O_2_ solution, indicating the chemical bond change into S = O according to the literature. Astaxanthin and Nile red were purchased from Sigma (St Louis, MO, USA). Cisplatin was provided by Aladdin (Shanghai, China). Chlorpromazine (CPZ), filipin, nystatin and nocodazole were purchased from Sigma (St Louis, MO, USA), and EIPA was from MedChemExpress (Shanghai, China). ROS probes, Amplex UltraRed Reagent and 2ʹ, 7ʹ-dichlorofluorescein diacetate (DCFHDA), were obtained from Invitrogen (Carlsbad, CA, USA) and Sigma. Texas Red conjugated cisplatin was supplied by Ursa Bioscience (Abingdon, MD, USA). The FITC-Annexin V/PI double staining kit was obtained from BD Biosciences (San Diego, CA, USA). The GSH-Glo™ Glutathione Assay kit was obtained from Promega (Madison, WI, USA). The IL-6 ELISA kit was provided by R&D Systems (Minneapolis, MN, USA). Antibodies, including cleaved-caspase 3, cleaved-caspase 9, P53, cytochrome-C and α-tubulin, were purchased from CST (Danvers, MA, USA), and Myosin VIIa was from Proteus Biosciences (Ramona, CA, USA). 4-hydroxynonenal (4-HNE) was purchased from Abcam (Cambridge, MA, USA).

### Cell culture

HEI-OC1 (generated at House Ear Institute, Los Angeles, CA, USA), an inner ear cell line derived from the auditory organ of the transgenic mouse Immortomouse^™^, is a potential tool for studying the mechanisms of ototoxic drugs in vitro [30]. HEI-OC1 cells were cultured in Dulbecco’s Modified Eagle’s Medium (DMEM) and supplemented with 10% (v/v) fetal bovine serum (FBS) in a 10% CO_2_ incubator at 33 °C.

### Animals

Healthy female adult guinea pigs (weighing 200–250 g, with no otitis media) and mouse pups (C57BL/6 J, P0-3) were used. Throughout the study, all animals were maintained under standard laboratory conditions, housed under standard animal facility conditions with adequate food and water. All animal studies were approved and conducted in accordance with the guidelines of the Ethics Committee of the Shanghai Jiaotong University, School of Medicine (Shanghai, China).

### Preparation of astaxanthin-loaded ROS-responsive nanoparticles (ATX-PPS-NP)

ATX-PPS-NP was fabricated using a single emulsion and solvent evaporation method as described previously [15]. Thirty milligrams of PPS-PEG and 2 mg ATX were dissolved in 1 ml of dichloromethane solution, followed by pouring 3 ml of sodium cholate solution (3%, w/v) into the organic solvent. The mixture was sonicated at 260 W for 4 min (Scientz Biotechnology Co., Ltd., Ningbo City, China). The resulting oil/water emulsion was further diluted in 36 ml of 0.5% sodium cholate solution and stirred overnight to remove the organic solvent. ATX-PPS-NP were collected by centrifugation (11,000×*g*, 30 min) and washed twice to remove excessive emulsifier and unloaded ATX. FITC-PPS-NP were prepared in the same way except that PPS-PEG was replaced with FITC-PPS-PEG. NiRe-PPS-NP were loaded with the fluorescent probe Nile red. The NP were stored at 4 °C.

### Characterization

Particle size, polydispersity index (PDI) and z-potential values were measured by zeta potential measurement (Nano ZS90 Zetasizer, Malvern Instruments Co., Ltd., UK) equipped with dynamic light scattering (DLS). Encapsulation efficiency (EE %) and drug loading (DL %) of the ATX-PPS-NP were determined using liquid chromatography combined with mass spectrometry (LC–MS) as described previously [16].

### ATX stability

Absorbance measurements were performed to assess the stability of ATX loaded in NP [31]. Natural ATX in EtOH/DCM (% v/v; 60/40) was used as the standard for the ATX calibration curve (absorbance-concentration curve) based on Beer–Lambert law, under the maximum absorption (λmax) at 490 nm. At various time points (0, 7, 14 and 21 days), ATX-PPS-NP or ATX were diluted and dissolved in EtOH/DCM for the ATX concentrations measurements by microplate reader. The remaining ATX was normalized by dividing the concentration of ATX at day 0.

### ROS-responsive release profile in vitro

The release kinetics of PPS-NP in vitro was simulated by the fluorescence quenching of loaded Nile red, a special fluorescent probe which fluoresces strongly in a hydrophobic environment, but quenches when released into an aqueous phase where it is poorly soluble. Ten microliters of NiRe-PPS-NP was added to H_2_O_2_ solution diluted in artificial perilymph (0, 5 and 500 mM) to each well of a 96-well plate which was stirred and placed in a gas bath at 37 °C. The Nile red fluorescence was measured using a microplate reader (Tecan Spark, Tecan Group Ltd.) at an excitation/emission wavelength of 535 nm/612 nm at specific time points from 0 to 72 h.

To evaluate ROS-responsive drug release, H_2_O_2_ solution was used to stimulate ROS release. Briefly, NiRe-PPS-NP were subjected to 0 or 500 mM H_2_O_2_ solution at each ON phase for 24 h, and replaced by PBS for another 24 h for the OFF phase, circulating for a maximum duration of 7 days. At the end of each interval, Nile red fluorescence was measured by a microplate reader (Tecan Spark, Tecan Group Ltd.) at an excitation/emission wavelength of 535 nm/612 nm.

To further evaluate disintegration of ATX-PPS-NP in the ROS environment, transmission electron microscopy (TEM) was used to observe the morphological changes in PPS-NP. Briefly, ATX-PPS-NP were treated with 0.3% or 3% H_2_O_2_ solution for 24 or 48 h.

### ROS-responsive release profile—in HEI-OC1 cells

HEI-OC1 cells were seeded and treated with 60 μM cisplatin for 24 h, followed by 24 h-culture in NiRe-PPS-NP. After washing twice, nuclei were stained with Hoechst 33,342. H_2_O_2_ solution (200 μM) was used as a positive control. Fluorescent images were captured with a laser confocal microscope (LSM880, Zeiss, Germany).

### Cellular uptake assay

HEI-OC1 cells (5 × 10^4^ cells per well) were seeded in a 24-well plate and allowed to adhere overnight. The cells were then incubated with FITC-PPS-NP for 1, 3, 6, 12 and 24 h. The cells were washed with PBS, fixed with 4% paraformaldehyde (PFA) for 15 min and stained with Hoechst 33,342. The FITC fluorescence was examined using a laser confocal microscope (LSM-880, Zeiss, Germany). To quantify cellular uptake, cells were prepared as previously described in a 6-well plate and collected for flow cytometry (BD Biosciences, Fortessa, CA, USA).

To examine the endocytosis mechanism of PPS-NP, HEI-OC1 cells were pre-incubated with the following inhibitors based on previous studies [32] at nontoxic concentrations: 15 µg/ml of chlorpromazine (CPZ), to inhibit clathrin-mediated endocytosis; 100 μM of EIPA (MCE), to block macropinocytosis and phagocytosis; 6 µg/ml filipin and 50 µg/ml of nystatin, to inhibit caveolae-mediated endocytosis; and 2 µg/ml nocodazole, to disrupt microtubules. The inhibitors were added to the cell culture medium or incubated for 45 min at 4 °C prior to addition of the FITC-PPS-NP suspensions for another 3 h. Subsequently, the cells were washed and collected for flow cytometry (BD Biosciences, Fortessa, CA, USA).

### ROS-responsive accumulation of PPS-NP

#### In HEI-OC1 cells

HEI-OC1 cells were cultured in dishes for confocal images. The cells were pretreated with CDDP (60 μM) or free medium for 24 h, and additionally co-incubated with Amplex UltraRed Reagent and FITC-PPS-NP. Immediately, the cells were imaged with a laser confocal microscope (LSM880, Zeiss) in a time series, at 10 min intervals for up to 30 min. H_2_O_2_ solution (200 μM) was used as a positive control.

#### In cultured Organ of Corti

The organs of Corti were dissected from neonatal mice (postnatal days 1–3) and cultured in the medium as described previously [17]. Explants were co-incubated with Texas Red conjugated cisplatin and FITC-PPS-NP for 24 h. The tissues were then stained with Myosin VIIa and Hoechst 33,342. Fluorescent images were captured with a laser confocal microscope (LSM880, Zeiss).

### Protective effect in HEI-OC1 cells

#### Cell viability assay

Cell viability in different patterns of cisplatin and drug was examined using the CCK-8 assay (Dojindo, Japan). In brief, HEI-OC1 cells (1 × 10^5^ cells per well) were seeded in 96-well plates and incubated overnight. The cells were divided into six groups (ATX, PPS-NP, ATX-PPS-NP, administrated at the same ATX concentration of 1 μg/ml) as follows: (1) Control, (2) drug alone for 24 h, (3) cisplatin (60 μM) for 24 h, (4) pretreatment with drug for 4 h and withdrawal of the drug followed by treatment with CDDP for 24 h (pre-4 h), (5) pretreatment with drug for 4 h followed by a co-treatment with CDDP for 24 h (pre-4 h + co-24 h) and (6) direct co-treatment with drug and CDDP for 24 h (co-24 h). After washing three times, 100 μl of dissolved CCK-8 solution was added to each well and incubated for 2 h at 33 °C. The absorbance was measured using a microplate reader (Tecan Spark, Tecan Group Ltd.) at 450 nm. All data were normalized based on background intensity. Blank PPS-NP with the same concentrations were also examined in all cases with nanoparticles involved.

#### Cell apoptosis study

Cellular apoptosis was quantified by flow cytometry using an FITC-Annexin V/PI double staining kit (BD Biosciences, San Diego, CA, USA). HEI-OC1 cells were pretreated with ATX, PPS-NP and ATX-PPS-NP for 4 h followed by co-treatment with CDDP for 24 h. The cells were then rinsed, trypsinized and collected after centrifugation. The cells were resuspended in 100 μl of 1 × binding buffer containing 5 μl FITC-Annexin V and PI, incubated for 15 min at room temperature in the dark and then analyzed by flow cytometry (BD Biosciences, Fortessa, CA, USA). Both Annexin V + /PI − cells representing early-apoptotic cells and Annexin V + /PI + cells representing late-apoptotic cells were considered apoptotic. Blank PPS-NP at the same concentrations were also examined in all cases with nanoparticles involved.

### Molecular mechanism of the protective effect in HEI-OC1 cells

HEI-OC1 cells were divided into six groups: (1) Control, (2) CDDP, 60 μM, 24 h, (3–5) pretreated with ATX, PPS-NP and ATX-PPS-NP, respectively, for 4 h followed by co-treatment with CDDP for 24 h. Each group was included in the following experiments.

#### Intracellular ROS level

The intracellular ROS level was detected using a fluorescent dye, 2’,7’-dichlorofluorescein diacetate (DCFHDA). Non-fluorescent DCFH is converted into the highly fluorescent DCF by an intracellular oxidation. For the assay, cells were seeded in a 24-well plate overnight and treated with drugs as above. After rinsing twice, 10 μM DCFHDA was added and cultured for 30 min at room temperature in the dark. Images were acquired by laser confocal microscopy (488 nm/530 nm) and fluorescence intensities of randomly chosen cells were quantified.

#### Glutathione (GSH) and interleukin-6 (IL-6) assay

HEI-OC1 cells (1 × 10^5^ cells per well) were seeded in 96-well plates and incubated overnight, followed by drug administration as above. After 24 h, the medium was removed and the content of GSH was calculated using a GSH-Glo™ Glutathione Assay kit. The removed medium was collected for IL-6 detection using an ELISA kit, according to the manufacturer’s protocol.

#### Apoptosis-associated proteins

The levels of apoptosis-associated proteins were evaluated by western blot. HEI-OC1 cells were cultured and treated with drugs as above. After washing twice with ice cold PBS on ice and lysed in RIPA, the protein extracts were subjected to 12% SDS-PAGE and electrotransferred to PVDF membranes. This was followed by blocking and incubation with specific antibodies to cleaved-caspase 3, cleaved-caspase 9, P53 and cytochrome-C (α-tubulin served as the control standard). The relative intensity was quantified by ImageJ software.

### Protective effect in cultured Organ of Corti

The organs of Corti were cultured as above and divided into five groups: (1) Control, (2) CDDP, 60 μM, 24 h, (3–5) pretreated with ATX, PPS-NP and ATX-PPS-NP, respectively, at the same ATX concentration of 1 μg/ml, for 4 h and followed by co-treatment with CDDP for 24 h. After fixation, hair cells were labelled with Myosin VIIa and quantified in the apex, middle and basal turns. Fluorescent images were captured with a laser confocal microscope (LSM880, Zeiss). The numbers of surviving hair cells in 1 μm tissue of Organ of Corti were calculated, and the percentages of those were compared in groups.

### Protective effect in guinea pigs

#### Evaluation of RWM penetration

NiRe-PPS-NP were used to investigate whether PPS-NP could penetrate the RWM. Briefly, guinea pigs were anaesthetized with ketamine (60 mg/kg, IP) and xylazine (10 mg/kg, IP). NiRe-PPS-NP in gelatin sponge were placed on the RWM as previously reported [15], for 0.5, 2 and 6 h, respectively (n = 3). After fixation, the RWM was microdissected and prepared for confocal imaging according to the procedures described in previous studies [33]. A series of images of the RWM by the z-axis scanning model at 1-µm vertical intervals from the middle ear side to the scala tympani side were collected and mean fluorescence intensities (MFI) were calculated. The outer epithelium layer (OE), connective tissue layer (CT), and inner epithelium layer (IE) of the RWM z-stack series images were differentiated by cell morphology.

#### In vivo release profile of ATX

Guinea pigs were divided into the control and cisplatin-treated groups and intraperitoneally injected with PBS and cisplatin (12 mg/kg), respectively. At designed time points of 0.5, 2, 6 and 12 h after RWM application of ATX-PPS-NP in the control group and 2 h in the cisplatin group, the cochleae were dissected after euthanasia and perilymph was collected by aspiration using a sharp glass pipette inserted into the scala tympani from the RWM. Samples were stored at − 80 °C. The concentrations of ATX were quantified using LC–MS. After diluting the samples, a 30 µL aliquot of the sample was added with 150 µL IS (diclofenac, 100 ng/ml) in ACN. The mixture was vortexed for 10 min and centrifuged at 3200 g for 10 min and then vortexed for 10 min and centrifuged at 5800 rpm for 10 min. A 5 µL aliquot of the supernatant was injected for LC–MS analysis. The minimum concentration detected was 1 ng/ml.

#### Protective effect—Auditory Brainstem Responses (ABR) tests

To evaluate auditory function, ABR tests were recorded in response to pure tone stimuli (4, 5.6, 8, 11.2, 16, 22.6, 32 and 45 kHz) between 0 and 90 dB in 5 dB increments using a closed-field microphone. The acoustic signals were generated with a RZ6 signal processor and BioSig software (TDT, Alachua FL, USA). Thresholds were defined as the lowest stimulus level at which a response was observed.

Guinea pigs were administrated with ATX-PPS-NP (0.36 mg/ml, 7 μl), ATX (0.36 mg/ml, 7 μl) or normal saline (N.S.) on the RWM. One hour later, the animals were intraperitoneally injected with cisplatin (12 mg/kg). ABR tests were conducted before the operation and one or three days after CDDP injection.

### Mechanism of the protective effect in animals

Guinea pig cochlea samples were prepared as reported previously [34]. Briefly, mouse cochleae were quickly dissected from the temporal bone following rapid decapitation. After fixation, decalcification and progressive dehydration, the samples were embedded in paraffin. Mid-modiolar section samples were then cut at 5–7 μm thickness and mounted on glass slides. Paraffin sections were deparaffinized in xylene, rehydrated through an ethanol series, and stained with H&E. For immunostaining of 4-HNE in SGNs, cochlear sections were prepared as previously described [35] and imaged with an optical microscope. R2, R3, R4 was defined as cochlear turns from apex to base. ImageJ software was used to measure the area of SGN regions. SGN density was defined by the number of SGNs/area of the SGN region. The integrated optical density (IOD) of 4-HNE staining in the SGN area was measured by ImageJ software.

### Statistics

All data are reported as mean ± standard deviation (SD). Analysis of Variance (ANOVA) with Bonferroni correction for multiple comparisons was used to determine treatment effects. p < 0.05 was considered significant.

## Results

### Characterization of ATX-PPS-NP

**Fig. 1:**
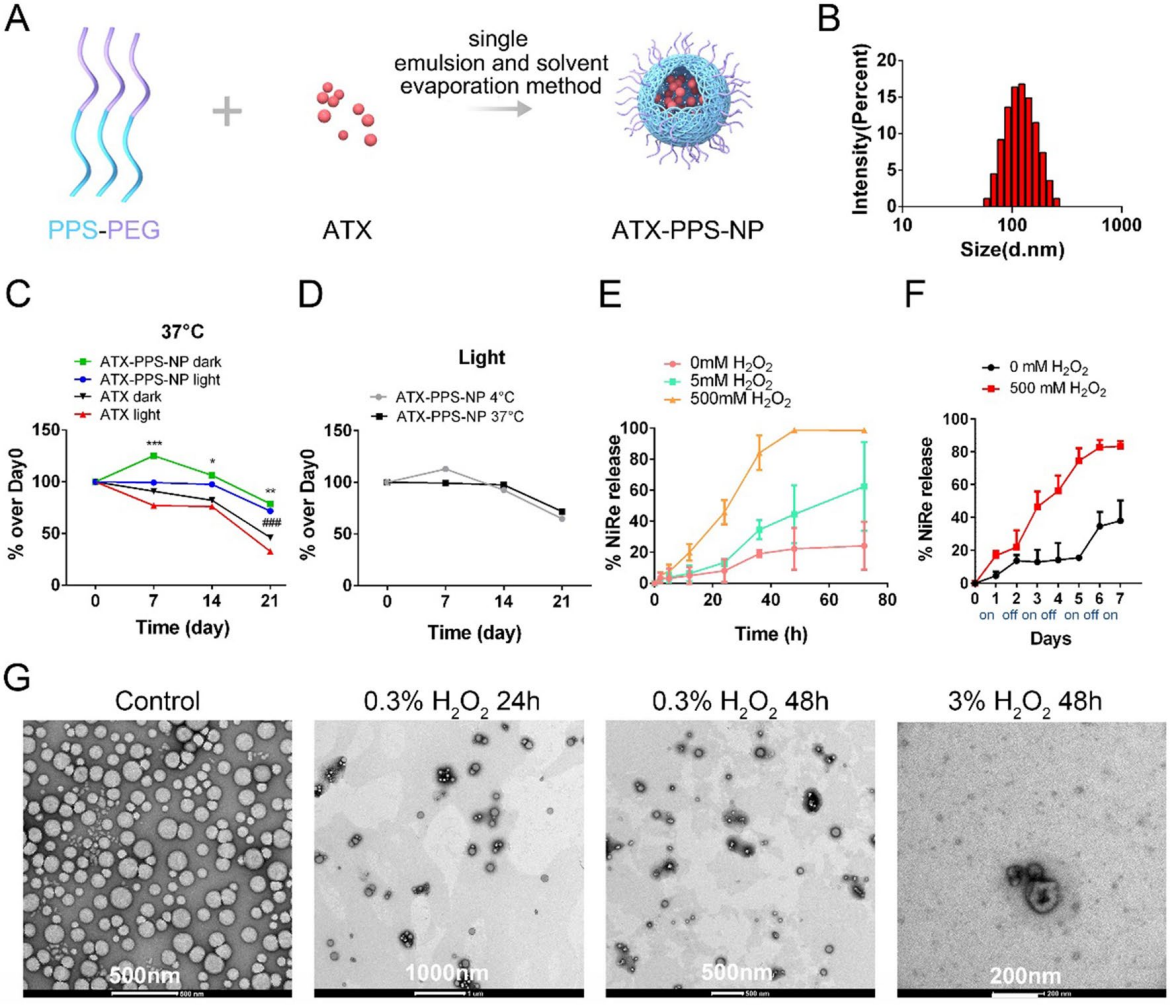
Physicochemical characterization of ATX-PPS-NP and in vitro ROS-responsive release. **A** The preparation of ATX-PPS-NP. **B** Gaussian distribution of the ATX-PPS-NP. **C**–**D** In 4 °C or 37 °C environments, the remaining percentages of ATX alone or encapsulated in PPS-NP in dark or light conditions within 21 days. ^*^p < 0.05, ^**^p < 0.01, ^***^p < 0.001 vs ATX dark, ^###^p < 0.001 vs ATX light. **E** In vitro Nile red (NiRe) release from NiRe-PPS-NP in various concentrations of H_2_O_2_ solutions. **F** In vitro release of NiRe with intermittent exposure (OFF/ON cycles every other day) to 500 mM H_2_O_2_ solution. **G** Representative TEM images of ATX-PPS-NP in control and various oxidation groups

The physicochemical characteristics of nanoparticles were validated (Fig. 1) by measuring particle size, PDI and z-potential values. As shown in Fig. 1B and Table 1, the particle size of PPS-NP and ATX-PPS-NP were 169.57 ± 7.87 nm and 182.27 ± 5.05 nm in diameter, respectively, and fitted the Gaussian shape line. A low PDI value (0.38 ± 0.02) also indicated a relatively uniform distribution of nanoparticles. Encapsulation efficiency (EE %) and drug loading (DL %) were 68.07% ± 3.02% and 4.31% ± 0.22%, respectively. Moreover, PPS-NP and ATX-PPS-NP were positively charged at 10.86 ± 3.27 mV and 14.73 ± 0.87 mV, respectively.

**Table 1:** Particle size, zeta potential, PDI values, drug loading (DL%) and encapsulation efficiency (EE%) of PPS-NP and ATX-PPS-NP

Nanoparticles (NP)	Particle Size (nm)	Zeta potential (mV)	PDI	EE %	DL %
PPS-NP	169.57 ± 7.87	10.86 ± 3.27	0.29 ± 0.03	/	/
ATX-PPS-NP	182.27 ± 5.05	14.73 ± 0.87	0.38 ± 0.02	68.07 ± 3.02	4.31 ± 0.22

We investigated the stability of ATX-PPS-NP by measuring the remaining concentrations of ATX at different time points (day 0, 7, 14, 21) under light or heat stimulation. As shown in Fig. 1C, ATX-PPS-NP significantly improved the stability of ATX on days 7, 14, and 21 under 37 °C with or without light (dark), characterized by the remaining concentration of ATX which was notably higher in the ATX-PPS-NP group than in the ATX group. Furthermore, on day 21, ATX-PPS-NP preserved a greater amount of ATX, 78% and 71%, respectively, in dark and light environments, compared with 46% and 32% in free ATX groups (p < 0.01, p < 0.001). Temperature had no impact on ATX stability when encapsulated in PPS-NP under light (Fig. 1D).

### ROS-responsive release profile in vitro

The ROS-responsive release from PPS-NP was assessed by the loss of fluorescence of encapsulated Nile red (NiRe) (Fig. 1E, F). Specifically, at each time point, the fluorescence value which was subtracted from that of the sample prior to H_2_O_2_ addition was normalized by the control. Under 5 mM H_2_O_2_ oxidation, approximately 45% of NiRe was released over 48 h, double higher than 20% released from the control group, while NiRe released from NiRe-PPS-NP oxidized by 500 mM H_2_O_2_ was approximately 100%. Moreover, NiRe release at each time point was higher in the oxidation group than in the control. These results indicated that ROS could quickly trigger the disintegration of NiRe-PPS-NP at an early stage and the encapsulated drug was released in a dose-dependent manner upon exposure to H_2_O_2_. To illustrate the smart release of encapsulated drugs, NiRe-PPS-NP were intermittently exposed to 0 or 500 mM H_2_O_2_ solution OFF/ON cycles every other day. The slope of the release curve was steeper during the ON phases than during the OFF phases, indicating that “on demand” release could be achieved. Together, the release profiles demonstrated ROS concentration-dependent, on demand release of the loaded drugs from PPS-NP.

To further evaluate disintegration of ATX-PPS-NP in the ROS environment, TEM was used to observe the morphological change in PPS-NP. As shown in Fig. 1G, PPS-NP exhibited a spherical structure with an average diameter between 100–200 nm, in agreement with the size measurement performed by DLS. After oxidation with 0.3%-3% H_2_O_2_ solution for 24–48 h mimicking ROS stimulation, NP progressively disintegrated and fused, suggesting ROS-responsiveness of PPS-NP.

### Cellular uptake and endocytosis mechanism

**Fig. 2:**
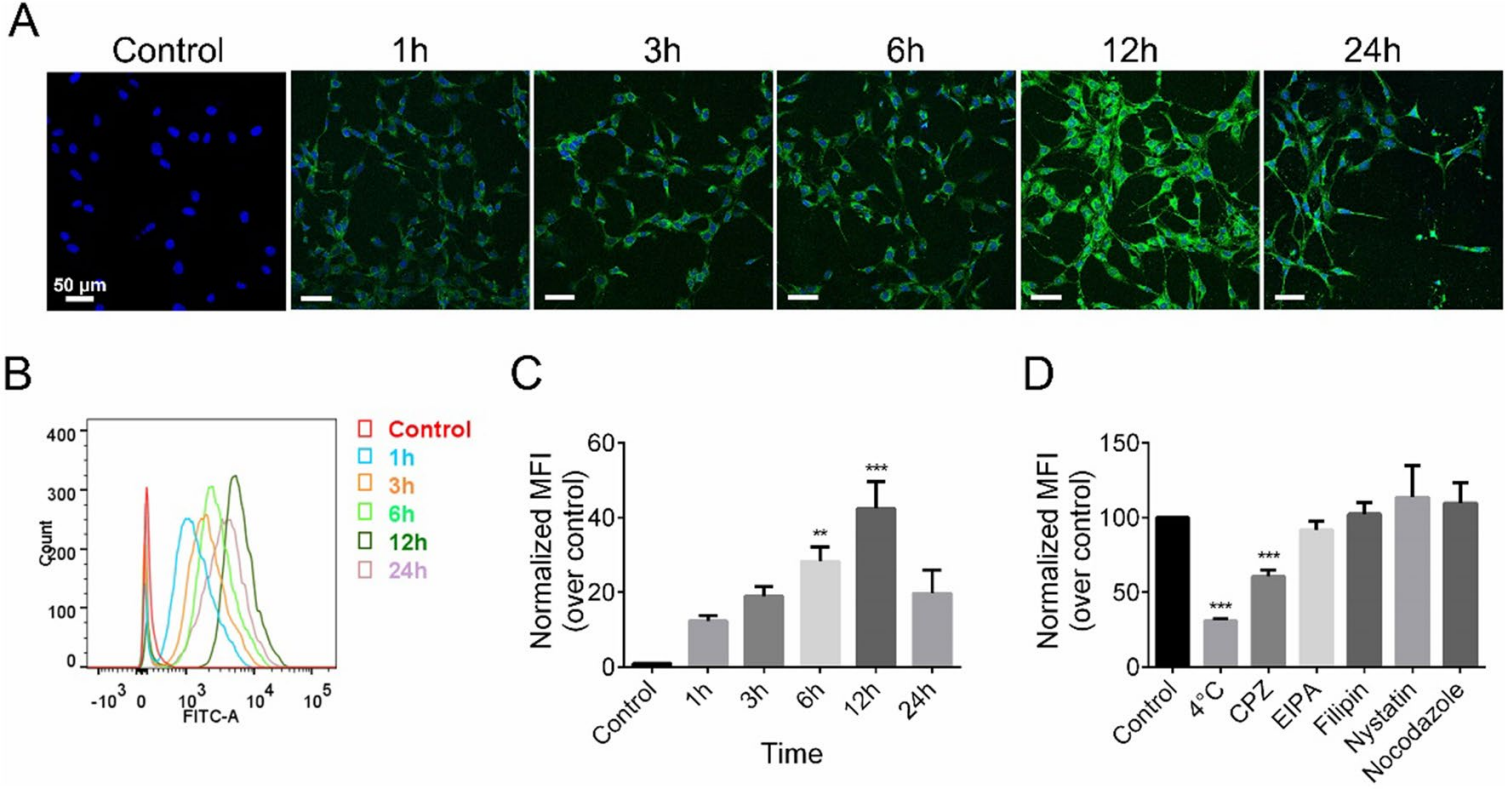
In vitro cellular uptake of FITC-PPS-NP. **A** Confocal images demonstrated cellular uptake of FITC-PPS-NP for various periods of time (1, 3, 6, 12 and 24 h) in HEI-OC1. **B**, **C** Flow cytometry assay of cellular uptake and quantifications of MFI of FITC. **D** Endocytosis mechanism of PPS-NP. ^**^p < 0.01 vs control, ^***^p < 0.001 vs control

To investigate the endocytosis of PPS-NP and the underlying mechanism, we fabricated FITC-PPS-NP as mentioned above, where FITC was used as a tracer fluorescein in the formulation of PPS-NP. Confocal laser scanning microscopy and flow cytometry were performed to observe and quantify the uptake profile in HEI-OC1 cells, respectively. As shown in Fig. 2A–C, cellular uptake showed a time-dependent increase between 1–12 h. After reaching the peak at 12 h, the uptake activity decreased at 24 h.

To elucidate the endocytic pathways of PPS-NP, we studied the effects of selective inhibitors and low temperature (4 °C) on cellular uptake. Of the inhibitors studied, treatment with CPZ led to significantly reduced fluorescence, suggesting that clathrin-mediated endocytosis may contribute to the uptake of PPS-NP in HEI-OC1 cells (Fig. 2D), in line with the size of particles (about 100 nm in diameter) internalized in the clathrin-mediated pathway [36]. Furthermore, low temperature (4 °C) also decreased the internalization of FITC-PPS-NP, suggesting energy-dependent endocytosis.

### ROS-responsive accumulation of PPS-NP in HEI-OC1 cells and cultured Organ of Corti

**Fig. 3:**
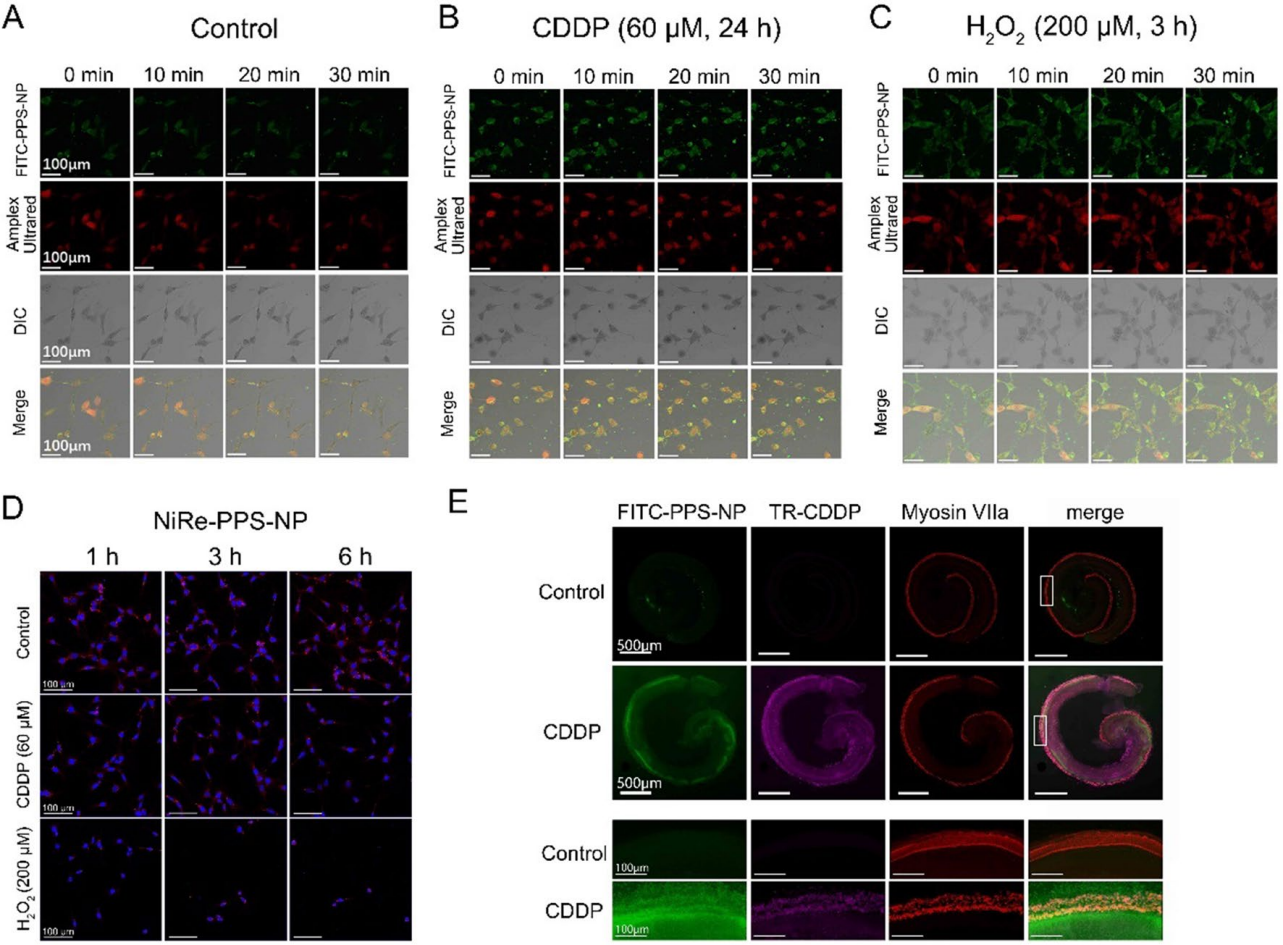
ROS-responsive accumulation and release profile in HEI-OC1 cells and cultured Organ of Corti. **A**–**C** Live cell imaging of cellular uptake of FITC-PPS-NP within 30 min under CDDP (60 μM, 24 h) stimulation, H_2_O_2_ (200 μM, 3 h) as a positive control. **D** Confocal images showed the Nile Red fluorescence changed at various time points in control or CDDP (60 μM) treated HEI-OC1 cells, H_2_O_2_ (200 μM) as a positive control. **E** Representative confocal images of cochlear explants. Texas Red (TR) and FITC fluorescence respectively labeled CDDP and NP, while Myosin VIIa labeled hair cells

Firstly, we screened the optimal dose and time for the CDDP-treated HEI-OC1 cell model. The results showed that 60 μM CDDP for 24 h induced approximately 70% cell viability and a significant generation of ROS (Additional file 1: Fig. S2). Then we evaluated the ROS-responsive accumulation of FITC-PPS-NP. In Fig. 3A–C, HEI-OC1 cells treated with CDDP (60 μM, 24 h) were co-cultured with FITC-PPS-NP and Amplex Ultrared, which respectively trafficked intracellular locations of NP and labeled ROS generated, and H_2_O_2_ (200 μM, 3 h) was used as a positive control. Serial images of live cells demonstrated that FITC-PPS-NP progressively entered cells and approached where ROS were located, suggesting passive ROS-responsive accumulation of FITC-PPS-NP in vitro. A stronger green fluorescence was observed in the CDDP group compared with the control group, indicating that ROS promoted the uptake of FITC-PPS-NP.

Figure 3D showed the intracellular ROS-responsiveness of NiRe-PPS-NP. In HEI-OC1 cells, NiRe-PPS-NP were quickly internalized. After exposure to CDDP (60 μM), the red fluorescence was quenched, indicating ROS-triggered release of NiRe into the aqueous phase in response to oxidative stress, while H_2_O_2_ (200 μM) was used as a positive control.

In Fig. 3E, Texas Red and FITC fluorescence trafficked CDDP and NP, respectively, while Myosin VIIa labeled hair cells. Compared with a fairly weak FITC signal in the control group, the whole and detailed images of organotypic culture in the cisplatin group showed a stronger green fluorescence, mainly in the spiral limbus and hair cells, suggesting that cisplatin treatment promoted the uptake of NP. The merging signal of Texas Red and FITC indicated that FITC-PPS-NP targeted the specific sites damaged by cisplatin, where ROS seemed to be generated.

### Cytoprotective and anti-apoptotic activity of ATX-PPS-NP

**Fig. 4:**
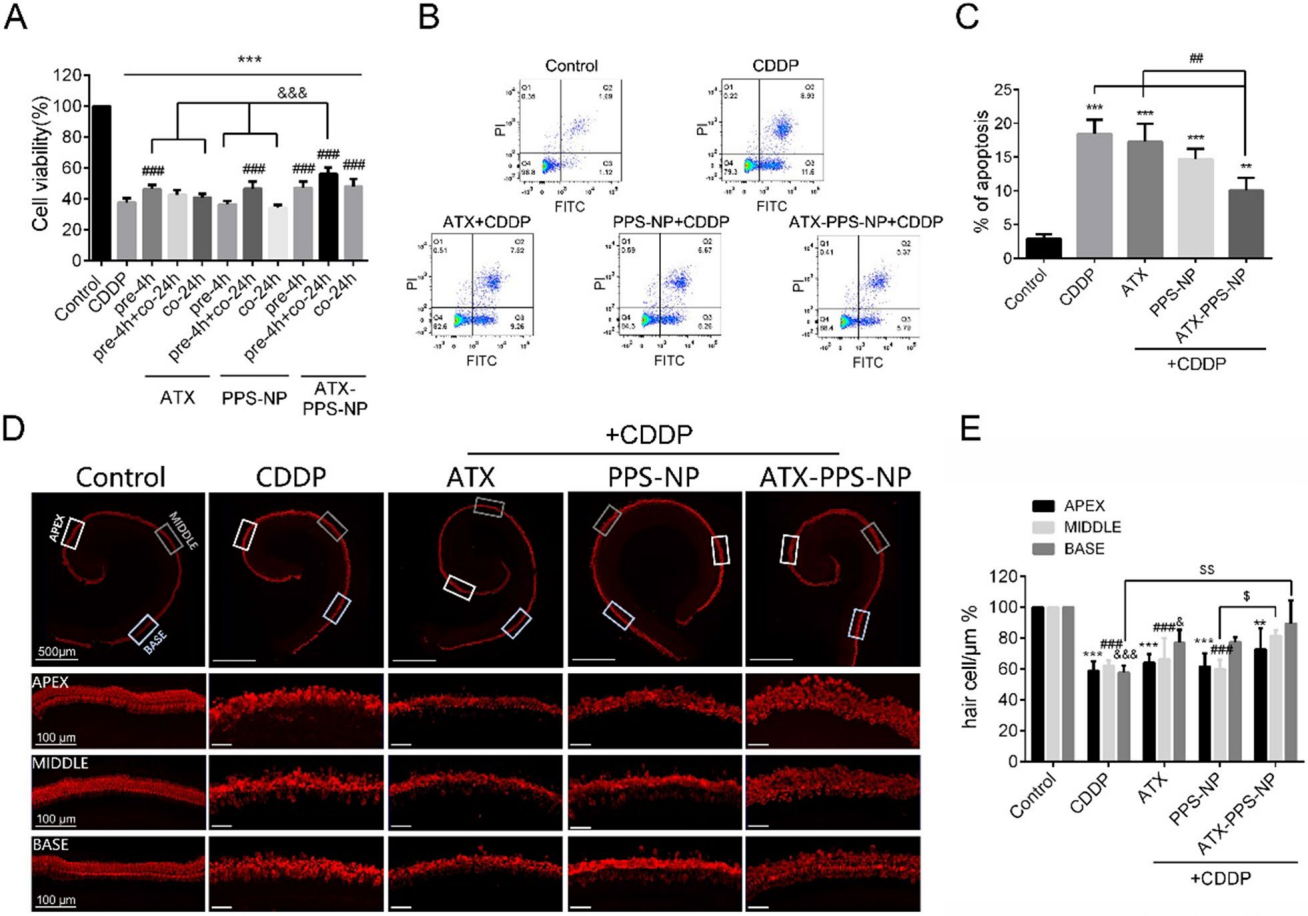
Cytoprotective and anti-apoptotic activity of ATX-PPS-NP against CDDP-induced toxicity in vitro. **A** A parallel comparison of cell viability in CDDP-treated HEI-OC1 cells with administrations of ATX (1 μg/ml), PPS-NP (drug free) and ATX-PPS-NP (1 μg/ml) in three types. ^***^ p < 0.001 vs control, ^###^ p < 0.001 vs CDDP, ^&&&^ p < 0.001 vs ATX-PPS-NP. **B** Flow cytometry showing the percentage of apoptotic cells labeled by Annexin V-FITC/PI double staining in ATX (1 μg/ml), PPS-NP (drug free) and ATX-PPS-NP (1 μg/ml) pretreated HEI-OC1 cells, followed by CDDP (60 μM, 24 h) exposure. **C** Quantifications of apoptotic cells. ^**^ p < 0.01, ^***^ p < 0.001 vs control, ^#^ p < 0.05, ^##^ p < 0.01, ^###^ p < 0.001 vs CDDP, ^&&&^ p < 0.001 vs ATX-PPS-NP. **D** Immunostaining of Myosin VIIa labeling hair cells in cochlea explants in control, CDDP (60 μM), ATX (1 μg/ml), PPS-NP (drug free) and ATX-PPS-NP (1 μg/ml) group. **E** Quantifications of hair cells in apex, middle and basal turn of cochlea explants. ^*, #, &^ p < 0.05 vs control, ^**, ##, &&^ p < 0.01 vs control, ^***, ##, &&&^ p < 0.001 vs control, ^$^ p < 0.05 vs CDDP, ^$$^ p < 0.01 vs CDDP

As illustrated in Fig. 4A, HEI-OC1 cells were treated with CDDP and different drugs, including ATX (1 μg/ml), PPS-NP (drug-free) and ATX-PPS-NP (1 μg/ml). Cell viability was significantly increased in the ATX pre-4 h group (p < 0.001) and drug-free PPS-NP pre-4 h + co-24 h group (p < 0.001), which was inhibited by CDDP. ATX-PPS-NP resulted in higher viability after pre-4 h + co-24 h of administration, up to 1.5-fold compared with that of the CDDP group. These results suggest that ATX-PPS-NP, combining ROS-consuming PPS-NP and antioxidant ATX, demonstrated an enhanced protective effect against cisplatin-induced cytotoxicity.

Annexin V-FITC/PI double staining was used to label apoptotic cells and the percentage of these cells was analyzed by flow cytometry (Fig. 4B, C). CDDP induced 18.43% of apoptosis, which was reduced by ATX-PPS-NP (p < 0.01), while no significant difference occurred in the ATX and drug-free PPS-NP group, confirming that ATX-PPS-NP exhibited notable efficiency in attenuating cell apoptosis.

As shown in Fig. 4D, E, the intact arrangement of hair cells was destroyed following CDDP administration and was partially restored by ATX-PPS-NP. To evaluate the protective effect of ATX-PPS-NP, hair cell counts were calculated. The results revealed that the CDDP group exhibited severe loss of hair cells and ATX-PPS-NP preserved hair cells in the basal turn of the organ of Corti explants.

### The mechanisms of the antioxidant, anti-inflammatory and anti-apoptotic effects of ATX-PPS-NP

**Fig. 5:**
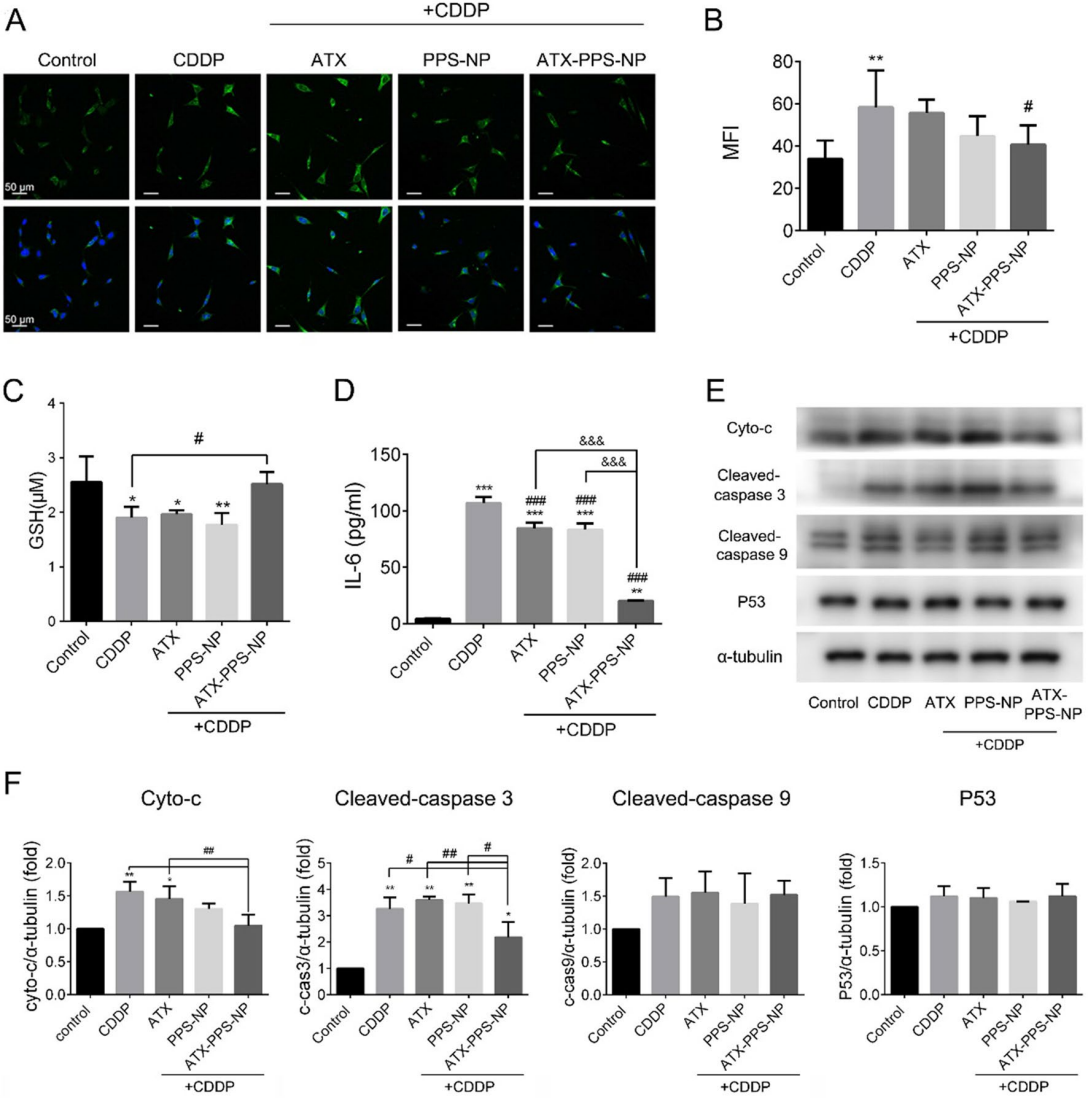
The mechanisms of antioxidant, anti-inflammatory and anti-apoptotic effects of ATX-PPS-NP.**A** Confocal images of intracellular ROS level stained by DCFHDA (green fluorescence) in ATX (1 μg/ml), PPS-NP (drug free) and ATX-PPS-NP (1 μg/ml) pretreated HEI-OC1 cells, followed by CDDP (60 μM, 24 h) administration. **B** Quantifications of MFI of DCFHDA. **C** GSH levels. **D** IL-6 levels. **E**–**F** The expression levels and qualifications of apoptosis-associated proteins evaluated by Western blot analyses. ^*^ p < 0.05, ^**^ p < 0.01, ^***^ p < 0.001 vs control, ^#^ p < 0.05, ^##^ p < 0.01, ^###^ p < 0.001 vs CDDP, ^&&&^ p < 0.001 vs ATX-PPS-NP

The underlying mechanisms of CDDP-induced ototoxicity are complicated, which resulted in the multifactorial protective effects of ATX-PPS-NP. We studied these effects from three aspects. Firstly, we explored the antioxidant effect. After screening the optimal drug treatment to reduce ROS production, pre 4 h + co 24 h was selected for the following studies (Additional file 1: Fig. S3). In Fig. 5A–B, as demonstrated by a reduction in MFI of DCFHDA (green fluorescence), the level of ROS induced by CDDP decreased following treatment with ATX-PPS-NP (p < 0.05), suggesting the highest antioxidant efficacy of ATX-PPS-NP against CDDP-induced accumulation of ROS among the three drugs studied. On the other hand, neither the ATX nor drug-free PPS-NP groups displayed significant reduction in ROS, confirming that a combination of ROS-consuming PPS-NP and the ROS-scavenging compound ATX led to an enhanced antioxidant effect.

It is acknowledged that the excessive generation of ROS overwhelms the redox balance [5]. We then detected the GSH level to investigate the antioxidant efficacy of ATX-PPS-NP. As shown in Fig. 5C, CDDP significantly decreased GSH level, while ATX-PPS-NP restored GSH level to approximately that of the control group when compared to the ATX or PPS-NP group. This indicated that ATX-PPS-NP strengthened the antioxidant defense system by reducing the depletion of GSH contents.

Secondly, the accumulation of ROS has also been identified as an important mediator of the inflammatory response. To clarify the anti-inflammatory effect of ATX-PPS-NP, a cytokine assay (IL-6) was performed. Compared to the control group, a significantly higher level of IL-6 was detected in the CDDP group. Treatment with ATX-PPS-NP was effective in decreasing cochlear proinflammatory cytokine levels (Fig. 5D). By contrast, free ATX alone or blank PPS-NP showed no significant suppression of IL-6.

Lastly, in terms of protein expression involved in the process of apoptosis, we evaluated the protein level of cleaved-caspase 3, cleaved-caspase 9, P53 and cytochrome-C (Fig. 5E, F). The results demonstrated that the expression of cytochrome-C and cleaved-caspase 3 was significantly elevated by CDDP treatment and subsequently reduced by ATX-PPS-NP. However, cleaved-caspase 9 and P53 were unchanged. Cytochrome-C is an initial factor in the mitochondrial apoptotic pathway and caspase-3 serves as a converging point in intrinsic and extrinsic pathways. These results suggest that excessive ROS induced by CDDP triggered mitochondrial depolarization and the cytochrome-C release, activating caspase-3 and ultimately leading to intrinsic apoptosis, which was suppressed by ATX-PPS-NP.

Overall, ATX-PPS-NP had potent antioxidant, anti-inflammatory and anti-apoptotic effects on CDDP-treated cells in vitro, eventually leading to increased cellular viability and attenuation of cell apoptosis.

### Evaluation of RWM penetration and in vivo release profile

**Fig. 6:**
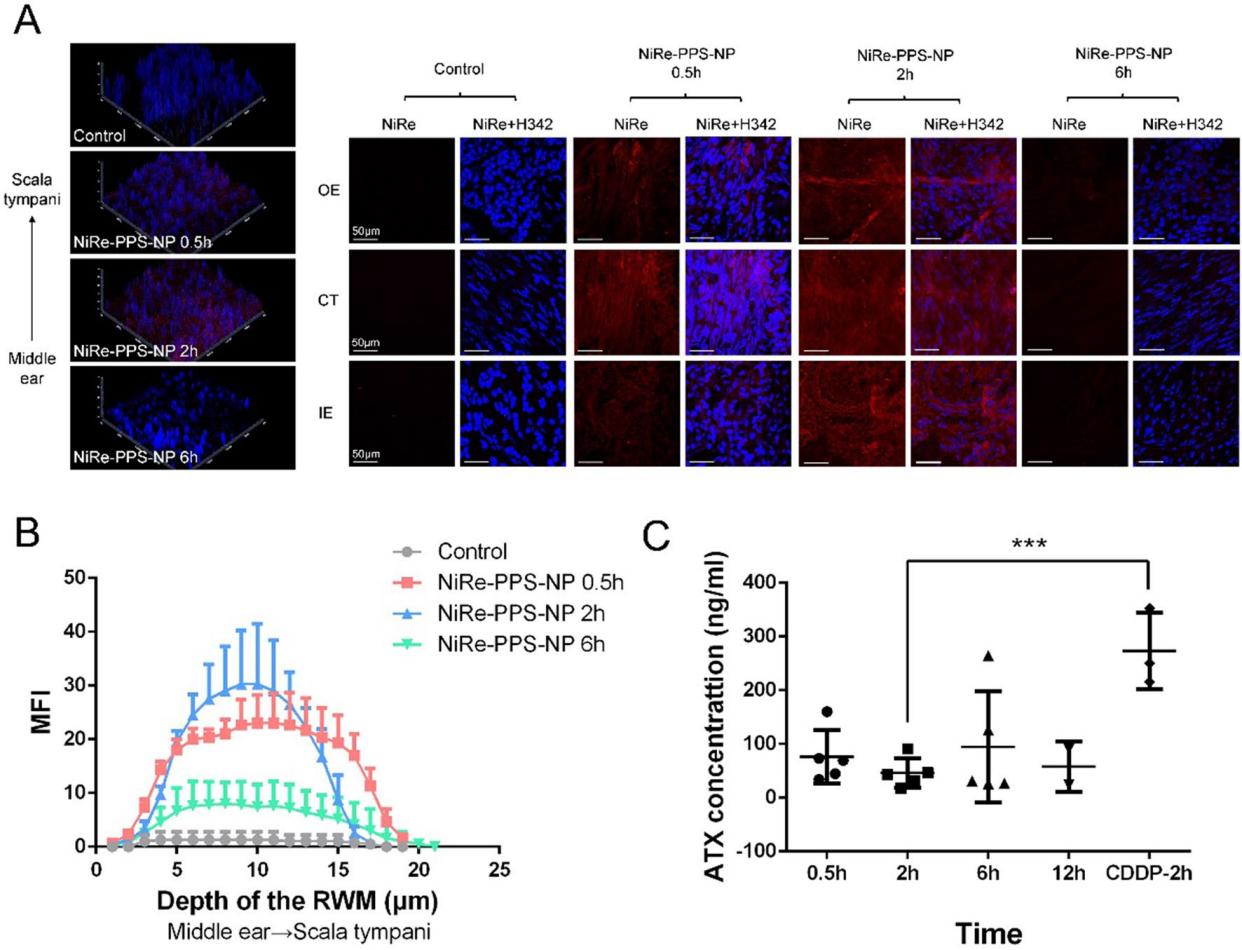
Evaluation of RWM penetration and in vivo release profile. **A** Fluorescent images of NiRe-PPS-NP penetrating three layers (OE, CT and IE) of round window membrane in control, 0.5, 2 and 6 h time points. **B** MFI of Nile Red along the RWM from middle ear side to scala tympani side. **C** ATX concentrations in control cochlear perilymph in various time points (0.5, 2, 6 and 12 h) and CDDP-treated ones (2 h). ^***^ p < 0.001

Previous studies on inner ear drug delivery systems mostly focused on their pharmacokinetics or cochlear distributions, but seldom on the interaction with the RWM. Here, we adapted a novel method reported by Chen et al. [33] to observe the RWM penetration process by NP. In detail, fluorescent images were captured in the three layers of the RWM (OE, outer epithelium layer; CT, connective tissue; IE, inner epithelium layer) at various time points after administration of NiRe-PPS-NP (Fig. 6A). As illustrated in Fig. 6B, the MFI of NiRe-PPS-NP increased quickly in the 0.5 h group, indicating the successful and rapid migration of NP from the middle ear side to the scala tympani side of the RWM. A significant increase in MFI was observed in the 2 h group, suggesting stable transport of NP into the inner ear. MFI in the 6 h group was reduced to a level lower than that in the 0.5 h group, which indicated that a majority of NP had penetrated the RWM into the cochlea.

To assess the penetrating capacity of ATX-PPS-NP through the RWM, we examined the ATX concentration in perilymph following the administration of a single dose of ATX-PPS-NP into the RWM. As shown in Fig. 6C, at 0.5 h after administration, the concentration of ATX which had diffused into the inner ear was 76.23 ± 9.74 ng/ml. This was sustained for almost 12 h. In contrast, ATX concentrations in the perilymph of CDDP-treated animals were much higher than those without CDDP damage, 272.97 ± 58.18 ng/ml vs. 46.00 ± 24.77 ng/ml (^***^ p < 0.001), suggesting the ROS-triggered drug release of ATX-PPS-NP in guinea pigs, consistent with the ROS-responsive drug release in vitro (Fig. 1E–G, 3D).

### Protective effects on auditory function in guinea pigs and underlying mechanisms

**Fig. 7:**
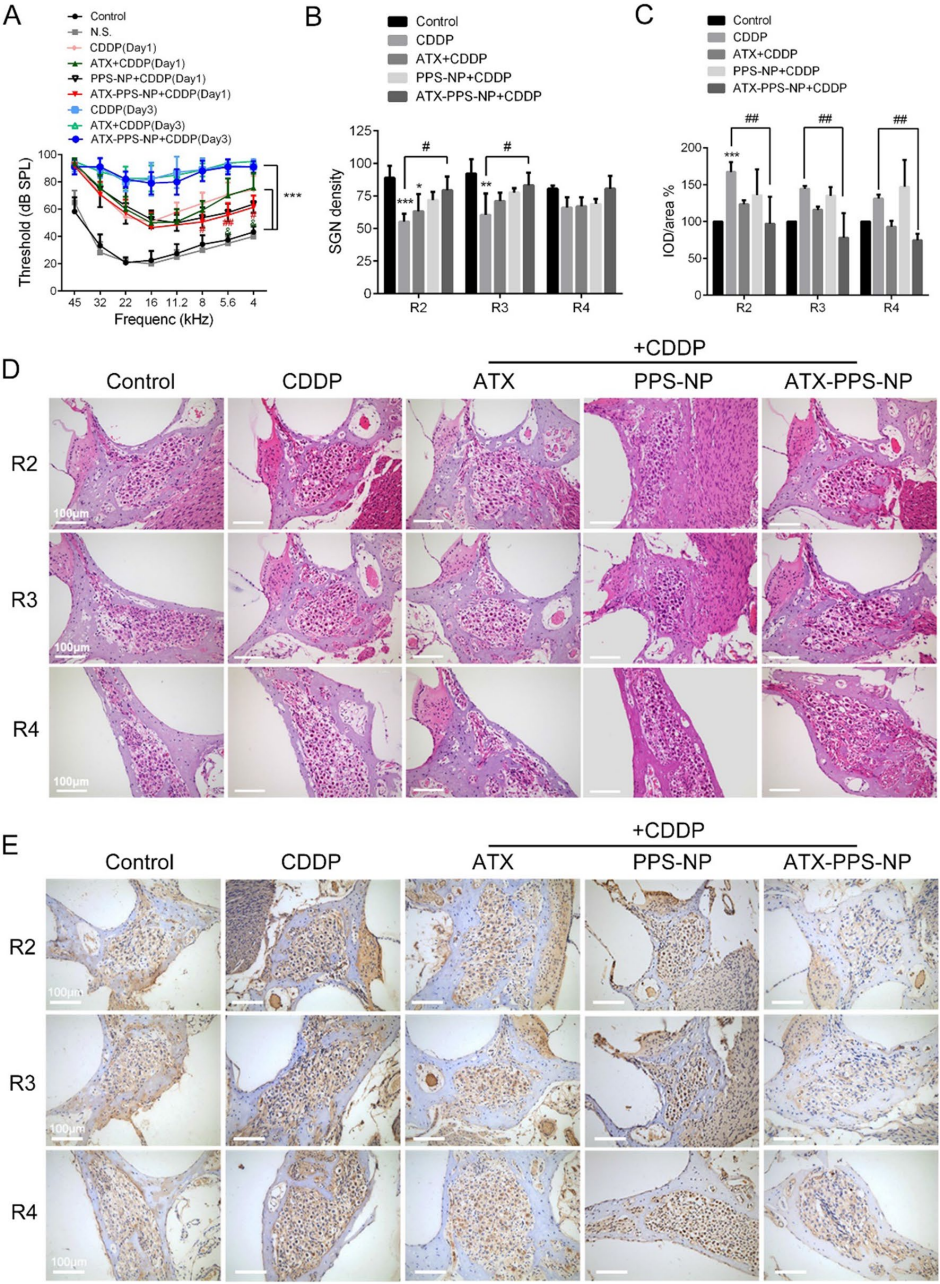
Protective effects on auditory function in guinea pigs and underlying mechanisms. **A** ABR thresholds of various groups. ^***^ p < 0.001 control vs CDDP (Day 1) and CDDP (Day 3), ^#^ p < 0.05 ATX-PPS-NP + CDDP (Day 1) vs CDDP (Day 1), ^##^ p < 0.01 ATX-PPS-NP + CDDP (Day 1) vs CDDP (Day 1), ^&^ p < 0.05 ATX-PPS-NP + CDDP (Day 1) vs ATX + CDDP (Day 1) **B** & **D**. **H**&**E** staining of SGNs and calculations of SGN density in 2–4 turns of guinea pig cochleae. ^*^ p < 0.05 vs control, ^**^ p < 0.01 vs control, ^***^ p < 0.001 vs control, ^#^ p < 0.05 vs CDDP. **C** & **E**. Immunohistochemistry of 4-HNE in SGNs and quantifications of integrated optical density (IOD) of 4-HNE staining. ^***^ p < 0.001 vs control, ^##^ p < 0.01 vs CDDP

Encouraged by the efficient cytoprotection of ATX-PPS-NP in vitro, the protective effect was then studied in vivo. Firstly, H&E staining verified that ATX-PPS-NP were well-tolerated, no inflammatory response was observed in the RWM (Additional file 1: Fig. S4). The surgical procedures used for RWM administration were further confirmed to be safe, based on no elevation in ABR thresholds (Fig. 7A). We then classified cisplatin-induced hearing loss into moderate and severe types according to the duration of CDDP treatment. It can be seen in Fig. 7A that ATX-PPS-NP administration protected against CDDP-induced moderate hearing loss by 13.57, 15.00 and 12.86 dB SPL at 4.0, 5.6 and 8.0 kHz, respectively, while no significant changes appeared in the unencapsulated ATX group. Taken together, these results verified that ATX-PPS-NP showed superior protection in HEI-OC1 cells, cochlear explants and animal models compared with equivalent ATX alone, revealing the enhanced antioxidant effect of ATX-PPS-NP. Additionally, no improvement in hearing was observed in animals with severe hearing loss, indicating that ATX-PPS-NP was only effective in protection against moderate hearing loss. We found that pretreatment of ATX-PPS-NP attenuated cisplatin-induced moderate hearing loss at low frequencies, with a negligible effect at high frequencies.

The present study revealed that focal vacuolization and nuclear degeneration were observed in cisplatin-treated SGNs, which was alleviated in the ATX-PPS-NP group (Fig. 7D). The SGN density was determined as the number of SGNs per unit area in the Rosenthal canal. As shown in Fig. 7B, ATX-PPS-NP preserved SGNs from destruction by cisplatin in the apical turn of the cochlea, consistent with the structural site where the protection of auditory function occurred. Animals treated with CDDP (Day 1) is an appropriate model for studying the mechanisms of ATX-PPS-NP in SGNs alone.

However, the number or morphology of hair cells and supporting cells were not influenced in CDDP-induced moderate hearing loss, and the thickness of the stria vascularis was not changed (Additional file 1: Fig. S5). The mismatching of protection targets between cochlear explants and guinea pigs could be the result of the specific damaging regions by different administration types. Direct exposure to culture medium containing cisplatin led to outer hair cell (OHC) loss, while systemically administrated cisplatin firstly caused SGN loss. In addition, functional impairments or subcellular changes which could not be obtained without performing ultrastructural level morphological analyses in OHCs may occur, but OHC count was unchanged on day 1 after CDDP administration.

To evaluate the in vivo antioxidant efficacy of ATX-PPS-NP, 4-HNE (a cytotoxic end product of lipid peroxidation, one of the markers for oxidative stress) was stained with 3, 3'-diaminobenzidine (DAB) for assessment of ROS generation. Figure 7C, E showed higher expression of 4-HNE in the cytoplasm of SGNs in the CDDP (Day 1) group compared to the control. Weaker expression of 4-HNE was observed in the ATX-PPS-NP group, with no changes in the ATX group. The quantifications of IOD/area demonstrated a reduction in 4-HNE in the apical and middle turns, which verified the strong ROS-scavenging ability of ATX-PPS-NP.

## Discussion

Up to now, cisplatin is an indispensable chemotherapeutic compound for a broad spectrum of solid tumors. However, its high therapeutic efficacy is often coupled with a potential ototoxicity with an average incidence over 60% [1]. With no pharmaceutical drugs approved by FDA, astaxanthin is considered as a promising candidate against cisplatin-induced ototoxicity due to its powerful antioxidant property, with ROS-scavenging capacity 6000 times higher than vitamin C, 550 times higher than vitamin E, 75 times higher than a-lipoic acid [37]. However, its bioavailability in inner ear is limited by low cochlear ATX concentration on account of its hydrophobicity, short half-life and susceptibility to chemical degradation under certain conditions (light, temperature, alkali, oxidation and isomerization) [38]. To overcome these limitations, PEGylation was applied in the fabrication of nanoparticles due to its effect in prolonging circulation half-life in serum by decreasing protein absorption and avoiding the formation of aggregates [39]. Here, we verified that the encapsulation of PPS-NP preserved the stability of ATX (Fig. 1C, D), in consonance with methods using microencapsulation with chitosan, polymeric nanospheres, emulsions and β-cyclodextrin in previous studies [40]. Previous studies indicated that size between 150 and 300 nm with a positive surface charge has advantages in permeating the RWM and entering the cochlea faster [41], which conferred ATX-PPS-NP advantages in providing sufficient ATX concentration in inner ear.

As previous studies have shown that ROS-responsive systems have the potential to achieve oxidation-specific drug release in a series of disease models [26–29], here, ATX-PPS-NP demonstrated ROS-responsive accumulation and enhanced drug release either in artificial perilymph, HEI-OC1 cell line or guinea pigs. Besides, the process of ROS-triggering disintegration of PPS-NP was also ROS-consuming, which facilitated an enhanced antioxidant effect when combining with ATX. Moreover, evidences indicated oxidative stress initiated the inflammatory process, resulting in the synthesis and secretion of proinflammatory cytokines [42], and activated mitochondrial apoptotic machinery. The reduction of IL-6 levels and the protein expressions of cytochrome-C and cleaved-caspase 3 in ATX-PPS-NP group suggested that an enhanced antioxidant efficacy further led to enhanced anti-inflammatory and anti-apoptotic effects on CDDP-treated cells. Eventually, it was verified that ATX-PPS-NP showed superior cellular protection in HEI-OC1 cell line, cochlear explants and guinea pigs compared with equivalent ATX. Thus, ATX-PPS-NP could reduce ATX doses and potentially minimize the side-effects caused by off-target effects [43], with protective efficacy guaranteed.

Outer hair cells of the organ of Corti, SGNs and stria vascularis are three major targets of cisplatin-induced ototoxicity. Time sequence studies [44, 45] exploring impairment processes at these 3 sites have verified that they run in parallel, other than secondary injuries dependent on another ototoxic process. A recent study found that a delay in ABR wave 1 latency which probably implied the damage to SGN mitochondria and myelination was the earliest functional and cellular changes after cisplatin treatment, indicating SGN as an early and direct damaging target in cisplatin-induced ototoxicity [46]. In the present study, the damaging target was determined by the analysis of histological and immunohistochemical staining of the basilar membrane and mid-modiolar sections. Notably, ATX-PPS-NPs increased the SGNs survival and preserved the greater morphology of SGNs in apical turns (Fig. 7B, D), with no noticeable changes of OHCs and SCs in Organ of Corti (Fig. S5). However, the lack of SGN cell lines and technological difficulty in the culture of Organ of Corti containing intact SGNs constricts the evaluation of the protection of ATX-PPS-NP on SGNs in vitro.

4-HNE is a cytotoxic end product of lipid peroxidation and can be eliminated by conjugating to GSH [35]. Here, we found that ATX-PPS-NP facilitated a reduced 4-HNE generation (Fig. 7C, E) and elevated level of GSH (Fig. 5C), suggesting the involvement of ATX-PPS-NP in the mitigation of lipid peroxidation and the further cell death induced by CDDP. As to the functional analysis, the pretreatment of ATX-PPS-NPs attenuated cisplatin-induced moderate hearing loss at low frequencies, with negligible effects at high frequencies (Fig. 7A). The reason why auditory function protection occurred in low frequencies is that, on one hand, cisplatin induces more severe hearing loss at high frequencies. On the other hand, drug efficacy is likely to depend on the intrinsic properties of the cochlea. For example, the capacity of spiral ganglion cells to respond to ROS challenges may vary along the tonotopic axis, with a higher SOD2 immunopositivity in SGNs located at the apex than those at the base [47].

## Conclusion

In the present study, we developed a novel drug delivery system combining antioxidant ATX and ROS-responsive/consuming nanoparticles (PPS-NP) to combat cisplatin-induced ototoxicity. ATX-PPS-NP were proven to be ROS-responsive in artificial perilymph, the HEI-OC1 cell line and guinea pigs. In addition, ATX-PPS-NP accumulated in ROS-specific sites and subsequently disintegrated for smart drug release. In HEI-OC1 cells, ATX-PPS-NP mitigated ROS level (DCFHDA) and inflammatory reaction (IL-6). Moreover, ATX-PPS-NP enhanced cell viability and alleviated cisplatin-induced mitochondrial apoptotic pathway by reducing the protein expression of cytochrome-C and cleaved caspase-3. In guinea pigs, RWM evaluation and LC–MS analysis verified the feasible penetration of ATX-PPS-NP into the inner ear. Based on these findings, we administrated ATX-PPS-NP on the RWM before CDDP injection and observed partial recovery of auditory function from moderate hearing loss. ATX-PPS-NPs increased the SGNs survival and mitigated the lipid peroxidation induced by CDDP. Therefore, this work provides an enhanced antioxidant therapy by combining ROS-responsive/consuming nanoparticles and ATX for cisplatin-induced ototoxicity.

## Supplementary Information

**Additional file 1: Figure S1.** Nuclear magnetic resonance ^1^H NMR spectra of PPS-PEG and FITC-PPS-PEG. **Figure S2.** Screening the optimal dose and time for CDDP administration in HEI-OC1. **Figure S3.** Screening the optimal application of drugs (ATX, PPS-NP, ATX-PPS-NP) in cell models. **Figure S4.** H&E staining of round window membrane (RWM). **Figure S5.** Structural changes of cochlea in CDDP (day1)-treated mice.

## Data Availability

Date and material are available for any research.
