# Supplementary material for: An enhanced antioxidant strategy of astaxanthin encapsulated in ROS-responsive nanoparticles for combating cisplatin-induced ototoxicity
Source: J Nanobiotechnology. 2022 Jun 10;20:268. doi: 10.1186/s12951-022-01485-8 (PMC9185887; doi:10.1186/s12951-022-01485-8)
Supplement: Supplementary file 1 — Additional file 1: Figure S1. Nuclear magnetic resonance 1H NMR spectra of PPS-PEG and FITC-PPS-PEG. Figure S2. Screening the optimal dose and time for CDDP administration in HEI-OC1. Figure S3. Screening the optimal application of drugs (ATX, PPS-NP, ATX-PPS-NP) in cell models. Figure S4. H&E staining of round window membrane (RWM). Figure S5. Structural changes of cochlea in CDDP (day1)-treated mice. [file 12951_2022_1485_MOESM1_ESM.docx]

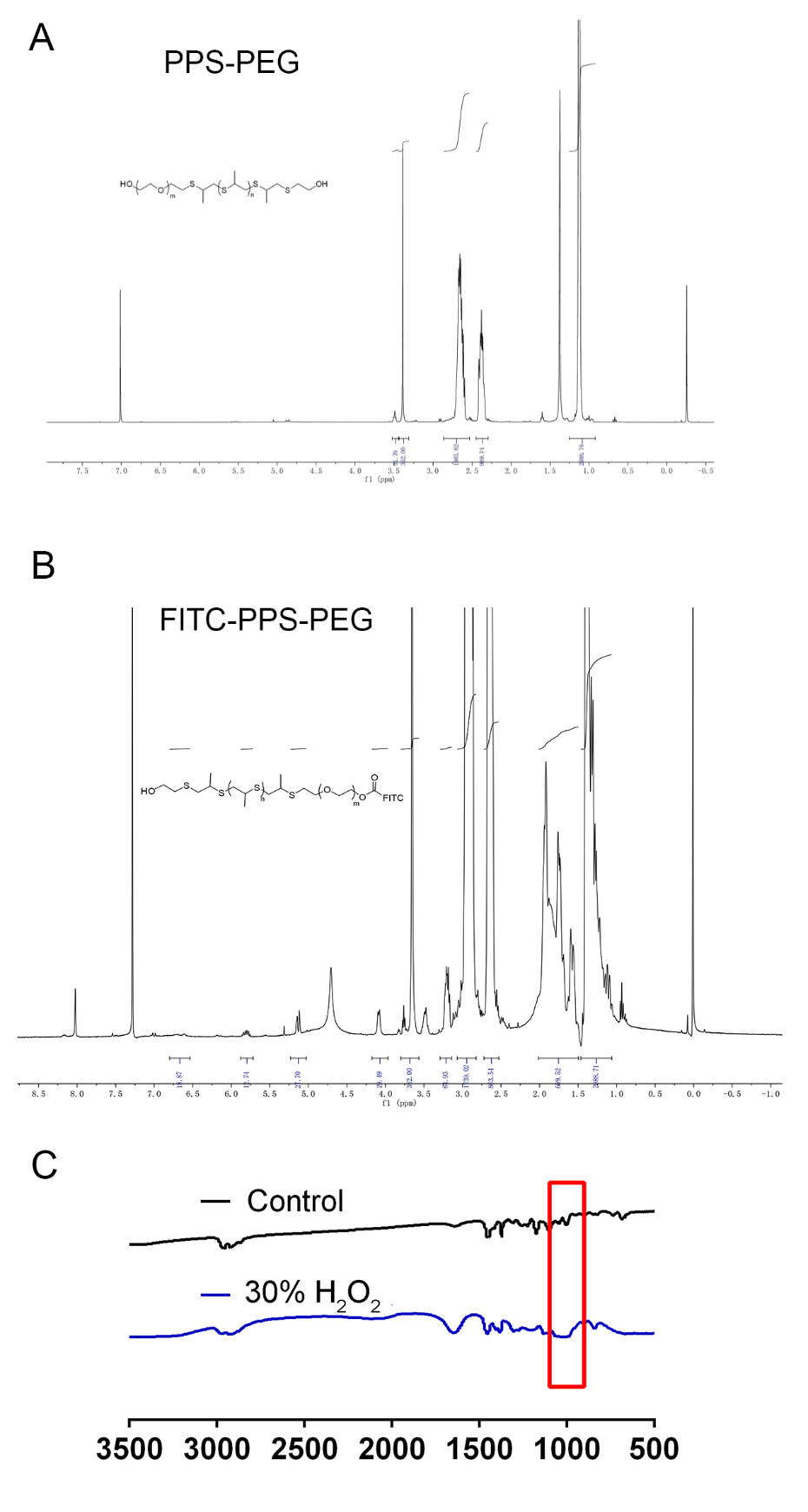


**Figure.S1. Nuclear magnetic resonance ^1^H NMR spectra of PPS-PEG and FITC-PPS-PEG.**

A-B. ^1^H NMR spectra of PPS-PEG and FITC-PPS-PEG C. FTIR spectra of PPS-PEG with/without H_2_O_2_ treatment.


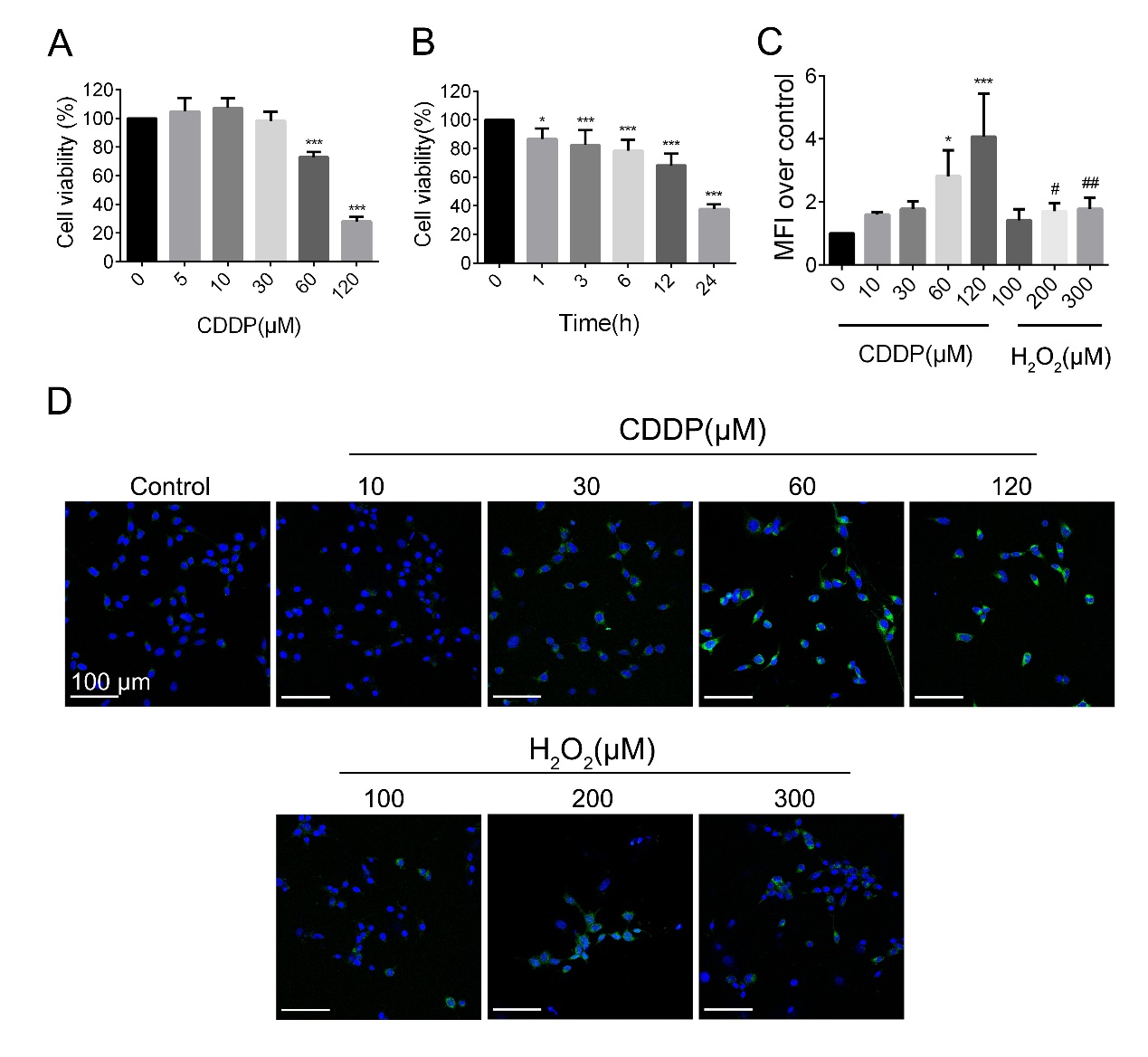


**Figure.S2. Screening the optimate dose and time for CDDP administration in HEI-OC1.** A-B. Cell viability of different doses (0, 5, 10, 30, 60 and 120 μM) and administration times (0, 1, 3, 6, 12 and 24 h) of CDDP. C. Quantifications of Mean Fluorescence Intensity (MFI) of DCFHDA by flow cytometry. D. DCFHDA staining (green) in HEI-OC1 cells treated with various concentrations of CDDP, H_2_O_2_ as a positive control for verification of the reliability of the ROS probe, DCFHDA. Nuclei were stained by Hoechst33342 (blue). * p<0.05 vs control, ** p<0.01 vs control, *** p<0.001 vs control.


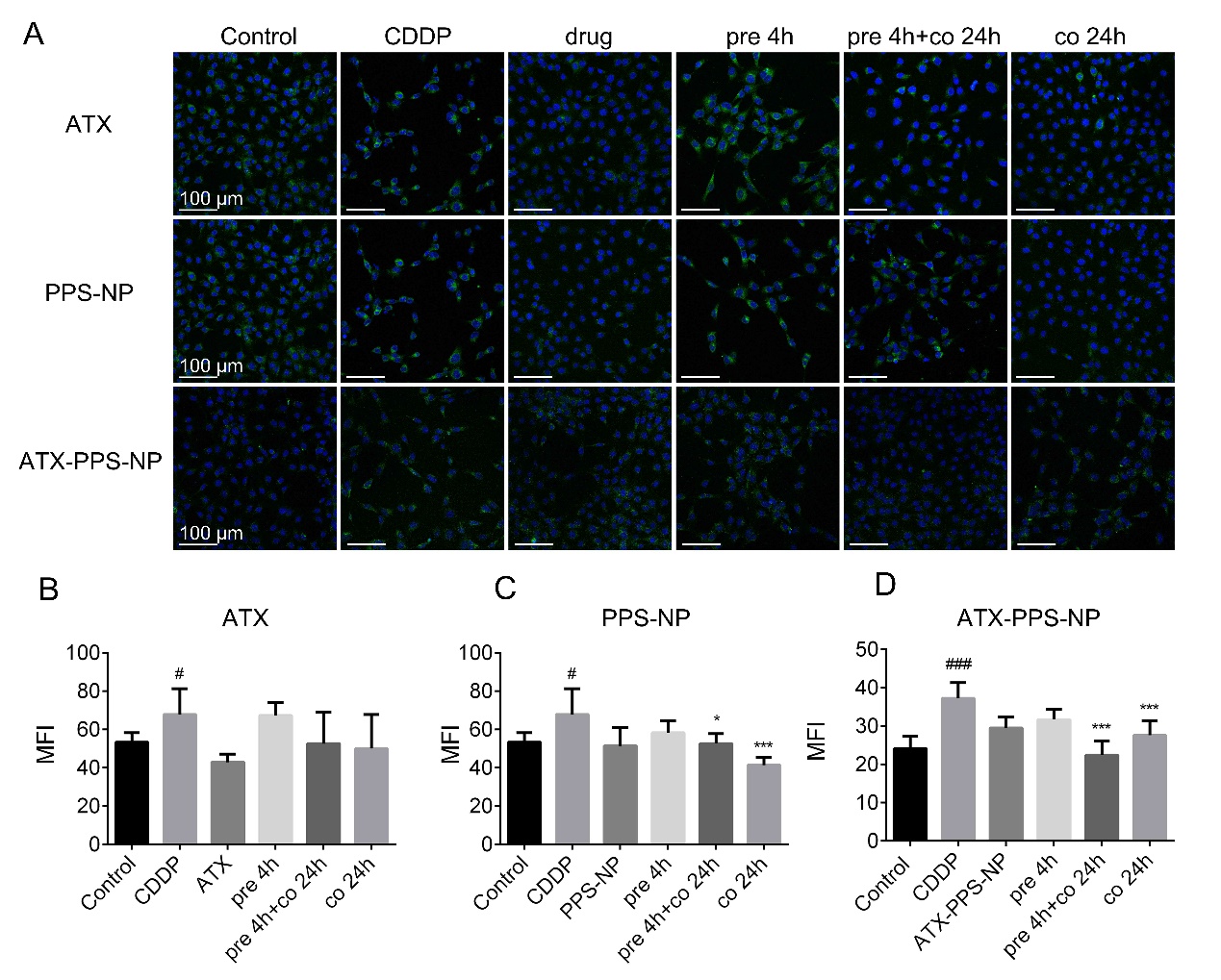


**Figure.S3. Screening the optimate application of drugs (ATX, PPS-NP, ATX-PPS-NP) in cell models.** HEI-OC1 cells were cotreated drugs and CDDP in the following ways: 1) pretreatment with drug for 4h and withdrawal the drug followed by treatment with CDDP for 24h; 2) pretreatment with drug for 4h followed by a cotreatment with CDDP for 24h; 3) directly cotreatment with drug and CDDP for 24h. A. DCFHDA staining (green) in HEI-OC1 cells treated with CDDP or drugs alone, or cotreated in these three ways above. Nuclei were stained by Hoechst33342 (blue). B-D. Quantifications of MFI of DCFHDA in A. # p<0.05 vs control, ### p<0.001 vs control, * p<0.05 vs CDDP, *** p<0.001 vs CDDP.


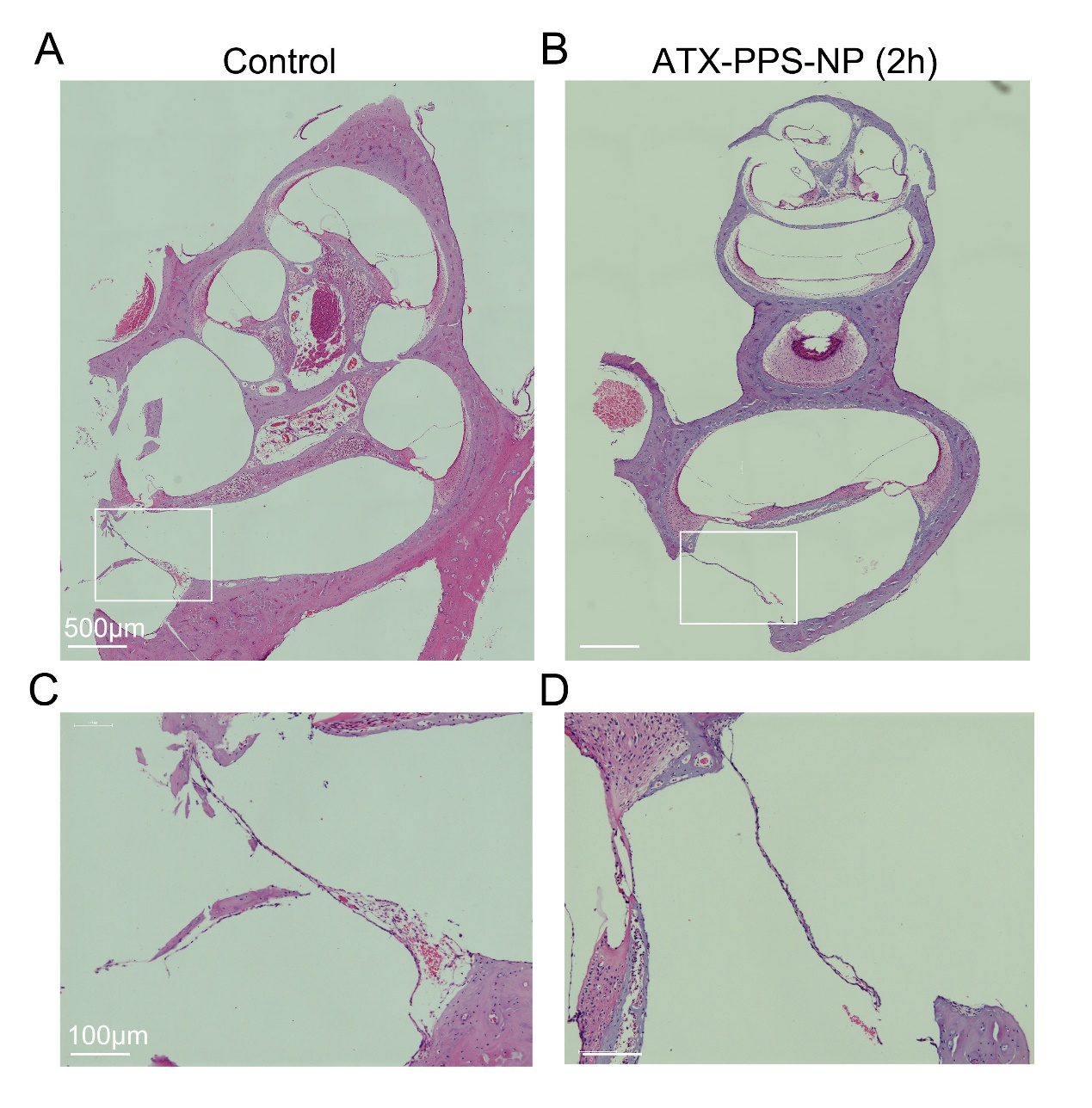


**Figure.S4. H&E staining of round window membrane (RWM).**

A-B. H&E staining of RWM in control and ATX-PPS-NP treated guinea pigs. C-D. Detailed images in the white rectangle in A-B.


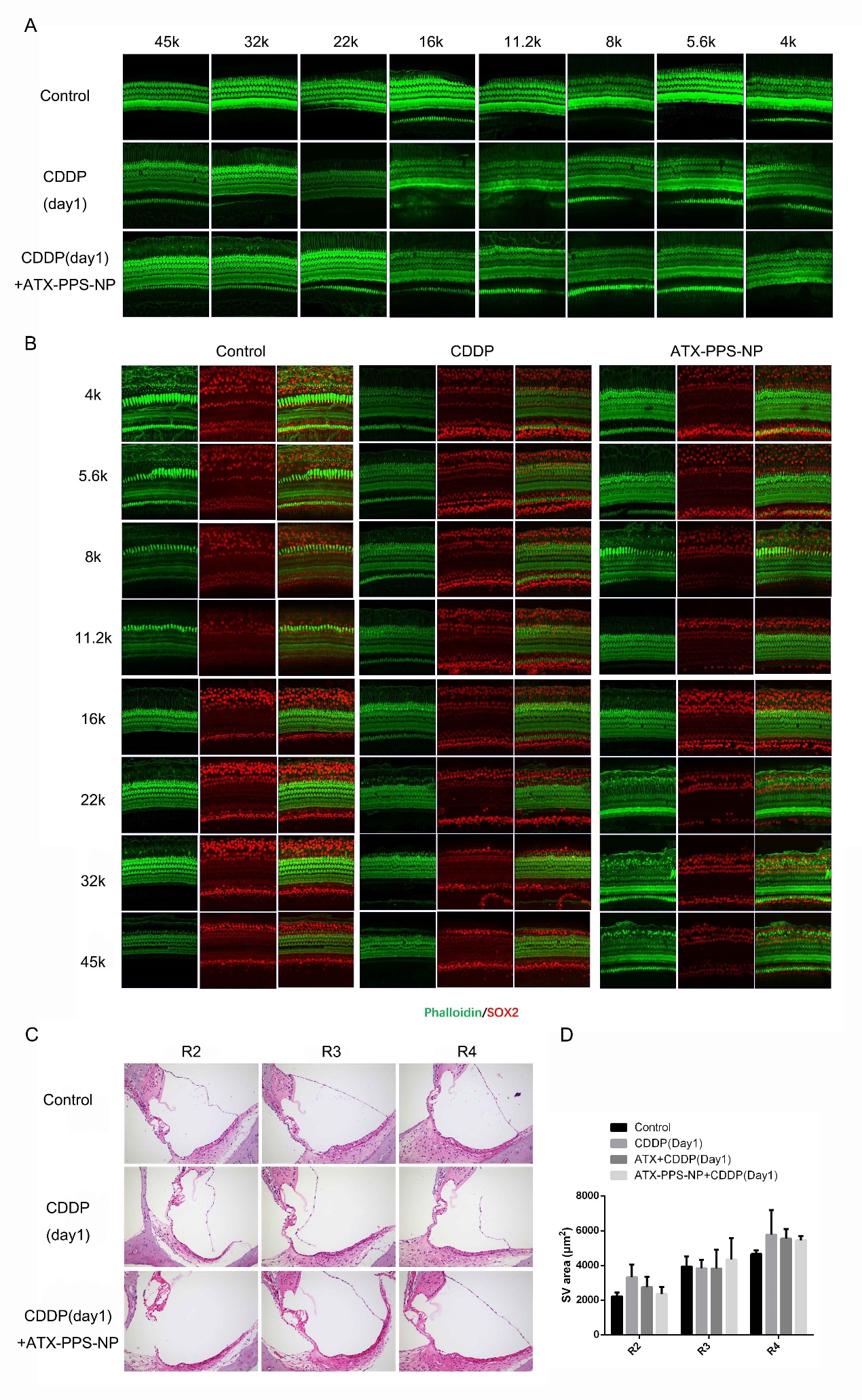


**Figure.S5. Structural changes of cochlea in CDDP (day1)-treated mice.**

A. Observation of hair cells in guinea pigs. Hair cells in control, CDDP (Day1) and ATX-PPS-NP+ CDDP (Day1) were labelled with phalloidin (green) and images were captured at 4, 5.6, 8, 11.3, 16, 22.6, 32 and 45kHz regions. B. Observation of supporting cells in guinea pigs. Hair cells and supporting cells in control, CDDP (Day1) and ATX-PPS-NP+ CDDP (Day1) groups were labelled with phalloidin (green) and SOX2 (red). Images were captured at 4.0, 5.6, 8.0, 11.3, 16, 22.6, 32 and 45kHz regions. C. Observation of Organ of Corti in guinea pigs. H&E staining of Organ of Corti in 2-4 rounds of cochlea in control, CDDP (Day1) and ATX-PPS-NP+CDDP (Day1) groups. D. Area of stria vascularis. Area of SV in control, CDDP, ATX+CDDP (Day1), ATX-PPS-NP+CDDP (day1) groups were quantified by ImageJ.
